# Surgery, Obstetrics and Gynecology

**Published:** 1904-11

**Authors:** William Warren Potter

**Affiliations:** Buffalo


### PROGRESS IN MEDICAL SCIENCE.

### Surgery, Obstetrics and Gynecology.

CONDUCTED BY WILLIAM WARREN POTTER, M. D., Buffalo.

### ABDOMINAL VERSUS VAGINAL HYSTERECTOMY.

JOHN B. DEAVER, (*American Journal of Obstetrics,* Jan., 1904, Vol. XLIX., p. 9). The author calls attention to the fact that irregular bleeding from the uterus, whether before, during, or after the menopause should always be thoroughly investigated. In addition, he believes that negative findings by the microscope, in cases of a curettement are to be accepted by the surgeon with great reserve, and should not be allowed undue weight in the cases which present unmistakable clinical symptoms, especially in a woman approaching, or past, the menopause. He believes that many of the cases diagnosticated as hemorrhagic endometritis would be the better for a complete hysterectomy. He does not wish to be understood as claiming that a negative finding by the microscope is to be considered as an argument against its value in diagnosis, but merely that the curette may have escaped the cancer area or that the latter was situated within the uterine muscle. The author limits the indications for hysterectomy more closely than certain other operators. He states that if the uterus be adherent and fixed in the pelvis, the broad ligament extensively infiltrated and the vaginal vault involved, operation is unadvisable. He does not believe in the removal of the greater portion of the vagina, as recurrence will surely occur and in all cases where such an extensive operation is required, new channels are also opened up for the extension of the disease. Such cases will do better by thorough curettage and cauterisation. He personally advocates the abdominal operation for all cases of carcinoma of the cervix, except in the presence of nephritis, senility or in very stout patients. He does not believe that it is necessary to dissect out the iliac glands, as the increased freedom from recurrence which is claimed, does not compensate for the additional mortality, and he calls attention to the fact that many enlarged glands are inflammatory and not cancerous. He believes in curettage of the cancer area and its cauterisation with pure carbolic acid before opening the abdomen.—*Therapeutic Review.*

---

PREGNANCY AFTER VENTRO-SUSPENSION AND VAGINO-FIXATION.

ONI, (*Annales de Gyn. et d'Obstetr.*, April, 1904,) from a review of the literature on this subject and his own experiences, finds that the ventro-suspension sutures must be placed on the anterior surface of the fundus uteri below the insertion of the tubes in order to avoid trouble in later confinements.

Vagino-fixation is to be done only in women past the menopause. Duhrssen's low vaginal fixation is not attended by trouble during labor, but is also not as sure a means of preventing a return of the retroflexion.—*Saint Louis Med. Review.*

---

TOILET OF THE NEW-BORN INFANT.

THE following routine is recommended by Riva-Rocci, (*Gaz. Med. Ital.*, Jan. 31, 1902): (1) insert the little finger in the mouth as far as the larynx and remove any mucus or foreign body there; (2) with 1-in-5000 solution of perchloride and a tampon of sterilised wool wipe the outer surface of the eye-lids; with a second tampon rapidly moisten the conjunctival sac with a few drops of this solution, and carefully dry the external parts; as a rule, no reaction occurs beyond a slight redness for a few hours: (3) with a third tampon cleanse the anterior nares; gonococcus infection here is not uncommon and is very intractable; a nasal douche has been suggested, but has its risks, and the author considers the other method efficient; (4) the bath should be of pure water: alkalies, soaps, and disinfectants are all irritating to the infant's skin; the only addition permissible is Unna's superfatted soap; the bath should be at a temperature of 95° F., so as not to produce cutaneous hyperemia and consequent nerve symptoms (in one case only is a hotter bath permissible, namely—in an asphyxiated child in whom the ordinary means of stimulating respiration have failed; it should then be given at 100° F.); the bath may last ten or fifteen minutes, if needed for cleansing; (5) dry the child with warm cloths, but not too hot, a mistake often made; for rapid drying, sheets of absorbent cotton are excellent; (6) powder the child with a fine absorbent powder, perhaps sterilised; an excellent one is Venetian talc and powdered starch, of each 1¾ ounces, crystalised carbolic acid ¾ grain, essence of lemon 10 minims; the whole affair should be evenly powdered, avoiding excess at any part.—*Medical Age.*

### A CASE ILLUSTRATIVE OF THE UNCLASSIFIED TROUBLES OF WOMEN.

E. C. Savidge (*Medical Record,* Oct. 22, 1904,) publishes a case illustrating his standpoint in regard to conservative gynecology. Many features in this patient's condition seemed to render operation necessary, but a careful consideration of the case as a whole and the institution of appropriate treatment yielded a restoration to health and enabled the woman to become a mother. The patient was a woman of thirty, highly neurotic and suffered twice every seven weeks from very profuse and painful menstruation, the attacks being so severe as to require the strongest anodynes and even chloroform. Physical examination showed an infantile uterus sharply anteflexed, with a narrow os, and both appendages large and tender. In addition, there were many "unclassified troubles," such as polyuria, cold feet, pelvic congestion, diarrhea, rapid heart, swelling thyroid, and great venous relaxation. The anemia was also severe. By treatment, consisting of rest in bed, massage, muscle wringing, resistant movements, feeding, free diuresis, and tamponage at and between menstrual periods, iron, intestinal disinfectants, and vasomotor constrictors the condition rapidly improved, and after forty days the cure was completed by a simple dilatation under primary anesthesia. Three years later the patient was delivered of a healthy child, and for the two years preceding pregnancy menstruation was painless and regular.

---

### THE CHINESE MANAGEMENT OF A TRANSVERSE PRESENTATION.

Dr. Anna R. Gloss, Pekin, China, states as follows: one day two men came to my house seeking a foreign doctor to go to the aid of a woman who was in labor. All they could tell me was that the "arm had come down and that the people were too poor to pay even the hire for cart for the doctor." Truly they were too poor. The house consisted of a single room, seven by eight feet. It was built of waste boards, paper windows and broken brick. A cotton curtain did duty as a door, even in the middle of winter. The kang or brick bed filled the entire room except a space two feet wide opposite the door. In this space, which had only a dirt floor, stood a table and a stove made from a discarded kerosene oil tin. Our wraps we left out of doors, and with difficulty we found a place inside to open up the obstetrical bag.

We found the patient on the kang dressed in her best clothes, half reclining in her mother's arms. A dying Chinaman is always dressed ready for the grave, so that the family need handle the dead body as little as possible.

The woman, we learned, had been in labor three or more days. The midwife had begun with the ordinary methods. Expensive incense had been burned under the mother's nose, a lighted candle had been placed in front of the vulva, the father had been sent upon the roof and his name repeatedly called in loud tones. All this to stimulate the child to come forth. Then the midwife sat behind the patient on the kang with her arms tightly clasped around her waist, making strong pressure from above, to compel the same desired end.

All should have gone well, but, unfortunately, the arm instead of the head came down. Then the midwife realised that she had a difficult case on hand. She procured some catsup from a neighbor, annointed the arm with it and repeated over it many times:

"Tzu, tzu, tsun, tsun, niang, niang,
Lung sheng, niang niang,
Kuai, Kuai tang tang tang ti."

Which, being interpreted, means, "Go back into the womb and come out properly." She then went home to dinner.

Returning some hours later and finding the arm still down, she went through the same performance, only using vinegar this time. Coming back again and finding no results from either the catsup or the vinegar, she tried the magic of salt and prayers. She did not use the deci-normal salt solution, but took the dirty brown salt used in their food. This also proving of no avail, she pulled upon the arm until it parted at the elbow, then upon the forearm until it also came away at the shoulder.

When there was nothing more within reach to pull upon, and pushing from above did no good, the midwife was in a dilemma. The pains had ceased and the patient was exhausted with loss of blood and want of sleep and food. At this point a neighbor's consultation was held in the court. Someone had heard of the foreign doctors, and two men kindly offered to go in search of one.

It was some hours later when we arrived on the scene, and in the meantime the family had given up all hope and dressed the sufferer ready for her coffin. Her pulse was so bad that with all possible haste we got her down on the kang by removing the old mother and the pile of pillows that supported her, took off the fine clothes, under which we found the pieces of dismembered arm, and the blood- and filth-soaked coarse paper, the only absorbent used in such cases. We brought her to the edge of the kang on a clean sheet, which we carry with us, and made a hasty wash-up. It is safe to say that this was the first washing of anything or anybody that had been done for three days. The child was soon delivered by turning. From its position and that of the placenta it must have been dead some time.

The vulva was so edematous and blue that it seemed the tissues must tear at touch, but they were not badly torn. The uterus was slow about contracting, but finally did so, and we left our patient to the tender mercies of her ignorant but anxious friends, warning them against making her sit up as soon as we were gone.

She quite properly had fever, but we were not called to the house again, though the husband came to us for medicine for her for ten days. Whether she finally lived or died we will never know, unless at some future time she needs to consult us. Then she will come and tell us that she is the woman whose life we saved.—*Chicago Clinic.*

